# A Review of Vision-Based On-Board Obstacle Detection and Distance Estimation in Railways

**DOI:** 10.3390/s21103452

**Published:** 2021-05-15

**Authors:** Danijela Ristić-Durrant, Marten Franke, Kai Michels

**Affiliations:** Institute of Automation, University of Bremen, Otto-Hahn-Alle NW1, 28359 Bremen, Germany; franke@iat.uni-bremen.de (M.F.); michels@iat.uni-bremen.de (K.M.)

**Keywords:** autonomous obstacle detection, on-board vision sensors, railways, traditional computer vision, AI-based vision

## Abstract

This paper provides a review of the literature on vision-based on-board obstacle detection and distance estimation in railways. Environment perception is crucial for autonomous detection of obstacles in a vehicle’s surroundings. The use of on-board sensors for road vehicles for this purpose is well established, and advances in Artificial Intelligence and sensing technologies have motivated significant research and development in obstacle detection in the automotive field. However, research and development on obstacle detection in railways has been less extensive. To the best of our knowledge, this is the first comprehensive review of on-board obstacle detection methods for railway applications. This paper reviews currently used sensors, with particular focus on vision sensors due to their dominant use in the field. It then discusses and categorizes the methods based on vision sensors into methods based on traditional Computer Vision and methods based on Artificial Intelligence.

## 1. Introduction

In 2018, 1666 significant railway accidents were reported in the European Union [1]. Out of this number, 939 accidents involved rolling stock in motion and humans (excluding suicides) and a further 442 were level crossing accidents including pedestrians. There were in total 853 casualties in these accidents, while another 748 people were seriously injured. As a result of various improvements in safety and in technology, the number of accidents has been decreasing over recent years with 563 fewer accidents in 2018 compared with 2010, a reduction of 25.3%. However, recent accident rates indicate that there is still scope for further improvement of railway safety. To date, the majority of work regarding obstacle detection in railways has concerned static obstacle detection systems at level crossings that are one of the highest risk elements of the railway system as they involve the potentially unpredictable behaviour of road and footpath users [2]. However, on-board sensor-based forward monitoring systems which provide continuous monitoring of the rail tracks in front of the train can complement the detection provided by static infrastructure such as level crossing monitoring systems, and so have the potential to significantly improve railway safety. This review paper presents an overview of the state-of-the-art techniques that allow on-board detection of obstacles on and near rail tracks.

In the last decade, significant developments in sensor technologies and Artificial Intelligence (AI), have led to increased research and development of on-board environment perception, including obstacle detection for road transport that has been reported in a number of papers including a number of review papers, for example [3,4,5]. Although railways are the other principal means of land transport, research and development of obstacle detection in railways has to date been much less intense than for road transport. Consequently, the number of published related works is significantly smaller than that for road transport. In [6], a review of vision-based on-board rail tracks detection methods is presented. However, the vision-based on-board obstacle detection is considered only from the perspective of pedestrian detection methods established for road vehicles. In addition, the authors in [6] do not include any overview/discussion of AI-based methods. Therefore, to the best of our knowledge, this is the first comprehensive review on on-board vision-based obstacle detection and distance estimation methods used in railway applications, which includes an overview and discussion of both traditional Computer Vision (CV) based methods and AI-based methods for rail tracks and obstacle detection in railways. Furthermore, to the best of our knowledge, this is the first paper discussing an overview of published works on estimation of distances between the on-board obstacle detection system and obstacles detected on or near the rail tracks.

### On-Board Sensor Technologies for Obstacle Detection and Distance Estimation in Railways

For a land transport vehicle to operate safely, accurate environment perception and awareness is fundamental, either by the driver alone or by the driver augmented by assistive technologies. Bearing in mind the advancements in environment perception for road vehicles over rail transport, and taking into account some similarities between these two means of land transport, it is understandable that the recently intensified research in obstacle detection for railways has built on research in the automotive field. That is, researchers and developers have applied and tested techniques that are known from the car driver assistance technologies to develop obstacle detection systems for rail transport.

An overview of on-board sensors for obstacle detection in railways is given in [7] and summarized here. These are typically the same as those used in automotive systems, and are active sensors such as LiDAR, radar and ultrasonic, as well as passive sensors such as stereo- and mono-standard RGB cameras and thermal cameras. The different sensors are analyzed with respect to usefulness of sensor types depending on weather/light conditions as well as with respect to distance detection range and cost. All sensors are characterized with some limitations under practical, real-world, conditions such as: limited use of LiDAR and ultrasonic sensors under heavy rain, no usability of standard cameras inside tunnels and in night conditions, and low contrast thermal images at high environmental temperatures. Independently of their limitations under real-world conditions, active sensors are used for direct precise measurements of obstacle distances. The advantages of cameras over active sensors are high data density and visual information that enables the detection of the boundaries of objects and the classification of these objects.

In order to use the positive characteristics of individual sensors, as well as to overcome the shortcomings of individual sensors, sensor fusion is a common solution for on-board obstacle detection (OD). One of the first OD systems consisting of an infrared camera and a laser rangefinder installed inside the train is presented in [8]. In [9], obstacle detection on a railway track by the combination of data from radar and camera sensors is presented. The same combination of radar and camera is used in [10], which describes how a multi-sensor system comprising a tele camera, a far distance radar, a survey camera and a near distance radar network were used for practical testing. A multi-sensor setup consisting of three video cameras and an infrared radar system, which monitors the track in front of a train, is presented in [11]. Different combinations of sensors, such as stereo vision, mono cameras, radar and laser, were implemented in the system presented in [12]. The paper focuses on the multi-camera system as one of the sensor subsystems. In order to fulfil accuracy requirements for reliable obstacle detection within 80 m ahead of the train, and to provide sufficient depth resolution to differentiate whether distant obstacles are on track or only nearby, the outer two cameras form a stereo camera system with a baseline of 1.4 m. The third camera, a color camera in the centre of the setup, primarily serves as a source for color information. An on-board railway object-detection system that automatically detects objects in front of the train in shunting mode is used in [13,14]. The system consists of an on-board camera that captures images for the proposed detection algorithm, and the distances between the detected object and train are measured using millimetre-wave radar. In [15], an 8-layer laser scanner, an RGB and a thermal camera were fused to allow a robust wagon and obstacle detection for the automation of a shunting locomotive. A multi-sensor system consisting of look-ahead sensors, video cameras (optical passive) and LiDAR (optical active) is presented in [16]. This system consists of a fixed short distance LiDAR (also called near LiDAR) and a two-dimensionally scanning long distance LiDAR (also called far LiDAR). These sensors use the time-of-flight principle and therefore provide a high longitudinal precision. The passive sensor unit is a multi-focal assembly of one survey fixed camera (also called short-range or near camera) and two far-range cameras (also called long distance or far cameras) with a pan device for the control of the cameras’ viewing angles.

Among all the commonly used on-board sensors discussed above, cameras are the ones that most precisely capture accurate data at high resolution. Like human eyes, cameras capture the resolution, minutiae and vividness of a scene with such detail that no other sensors such as radar, ultrasonic and lasers can match. Because of this, and as confirmed in the above-mentioned papers, cameras are in practice essential sensors in multi-sensors OD systems as only vision sensors can provide explicit rich visual information about the scene in front of the train. In addition, the fact that cameras are currently less expensive than other typical sensors for OD solutions has influenced the significant amount of research and development of vision-based on-board obstacle detection systems. Advances in computational platforms and vision algorithms have enabled the use of low-cost cameras to capture images in real time and perceive the surroundings of a rail vehicle, leading to a number of purely vision-based on-board OD systems being developed.

There are various possibilities for the use of on-board frontal cameras in trains published in the literature. Methods based solely on the use of cameras attached in the front of the train are presented in [17,18]. Research in [19,20] presents the application of a monofocal video camera, mounted behind the wind shield of the train, to improve the track localization quality. Train front cameras are also used in [21,22]. In [23], a cost-efficient vision system consisting of a camera that observes the area in front of a train is used for rail tracks detection in real time. In [24], a vision system consisting of a monocular thermal camera mounted on a train for detecting the rails in the imagery as well as for detecting anomalies on the railway is presented. In some published works, single camera systems are augmented to improve their data recording capability. In [25], a prototype system for railway object detection, installed in the cab of a train, is described; this system consists of a single camera that acquires images of the railway scene in front of the train and a near infrared laser mainly used to add illumination to the area the camera is directed at when the light is insufficient. A system in which a zoom lens camera is mounted on a pan–tilt unit so the camera image always tracks the rails is presented in [26].

A number of authors present the application of multi-camera systems and long-range cameras to advance environment perception performance. For example, in [27], various on-board camera models and lenses were used to extract rail tracks in short as well as in long distances. A vision-based system consisting of a high-resolution camera capable of detecting relevant objects within a distance of 500 m that is mounted on the body of the locomotive to have a clear view of the scene in front of the train is presented in [28]. In [29], the use of an ultra-telephoto lens camera mounted on train to monitor the status of the track 600 m (the braking distance) or more ahead of the train is investigated.

Bearing in mind the dominant use of vision sensors in on-board OD systems either as the sole sensor type or combined with different sensor types, and the principal role of vision sensors to provide rich visual information of the railway scene in front of a train, this review paper puts a particular focus on methods for obstacle detection and distance estimation that are vision-based.

The rest of the paper is organized as follows. Section 2 is divided into two main sub-sections describing two main groups of vision-based on-board OD methods, traditional CV based methods (Section 2.1) and AI-based methods (Section 2.2). Each of these two sub-sections is further divided for discussion of papers including rail tracks detection (Section 2.1.1 and Section 2.2.1), obstacle detection (Section 2.1.2 and Section 2.2.2) and estimation of distances between the train and detected obstacles (Section 2.1.3 and Section 2.2.3). Section 3 reviews papers that include OD evaluation tests in the following categories: tests on images/videos recorded in real-world operational environments, and tests on datasets found in the Internet. The paper ends with a discussion of some promising directions for future research (Section 4).

## 2. Vision-Based On-Board Obstacle Detection in Railways

Based on the used techniques for image features extraction, all methods for vision-based on-board obstacle detection can be divided into two main groups: methods based on traditional CV and methods based on AI. The first group has methods which use well-established CV techniques for both image segmentation (e.g., edge detection, corner detection or threshold segmentation), and for object recognition based on the extraction of so-called “hand-crafted” features. “Hand-crafted” features refer to properties extracted from an image (e.g., edges, corners, and shape descriptors of segmented image regions) using various methods that exploit the information present in the image itself. The AI-based methods in the second group are based on Machine Learning (ML) and in particular on Deep Learning (DL). In contrast to traditional CV methods that use “hand-crafted” image features to classify image areas as belonging to particular object classes, DL uses the concept of “end-to-end” learning of object detection, based on multi-layer artificial neural networks, in which the machine (computer) is given a dataset of images which have been annotated with what classes of objects are present in each image of the dataset. Deep layers in these complex neural networks act as a set of generic feature extractors that are, to some extent, independent of any specific classification task. This means that a DL-based method results in a set of image features learned directly from the observations of the input images. These features are in literature referred as “non hand-crafted” features as opposed to those extracted by traditional CV methods [30]. Independently of the type of the method, a primary function of an autonomous vision-based on-board railway OD system is to detect and classify the objects at a certain range to avoid collisions and to determine potentially hazardous situations during the train operation. Bearing in mind that the objects that are hazardous to regular functioning of a train are those which are on and near the rail tracks, a complete autonomous on-board system should include rail tracks detection and detection of objects on/near the rail tracks as well as estimation of the distance between the detected object and the train. However, to the best of our knowledge, until this time, there have not been any published works which include all these essential components. Rather, publications present only one or at most two of three essential functionalities. In addition, most related publications describe evaluation tests of only limited scope for the methods investigated, such as evaluation using only images available in the Internet, or on a limited number of real-world images recorded in restricted real-world railway environments. A small number of papers describe the evaluation of developed methods in real-world trials in operational railway environments.

The papers covered by this review describing solely traditional CV methods and describing solely AI-based methods are summarized in Table 1 and Table 2, respectively. Besides these two groups of papers, there are several papers that present hybrid systems which use traditional CV methods for rail tracks detection and AI-based methods for object detection. These papers are summarized in Table 3.

### 2.1. Traditional CV Methods

Bearing in mind that the main goal of OD in railways is the detection of objects (possible obstacles) on and near the rail tracks, almost all traditional CV methods include rail tracks detection for the purpose of identifying the image Region of Interest (ROI), within which the search for the objects that are hazardous to the train should be performed. Traditional CV obstacle detection algorithms are therefore mainly designed to find the rails up until an obstacle. In fact, some of the methods presented in the literature, as described in the section below, contain only rail tracks detection and do not explicitly consider obstacle detection at all; instead, they define the obstacle free region to be from the train to the first point where the rail track is no longer detected, either because of an anomaly on the rail tracks or because rails are outside the field of view. This is because these methods assume that finding the obstacle-free rail tracks region, more explicitly the extent of the continuous (non-interrupted) rail track region in front of the train, is sufficient for defining the collision free region.

#### 2.1.1. Rail Track Detection

**Figure 1:**
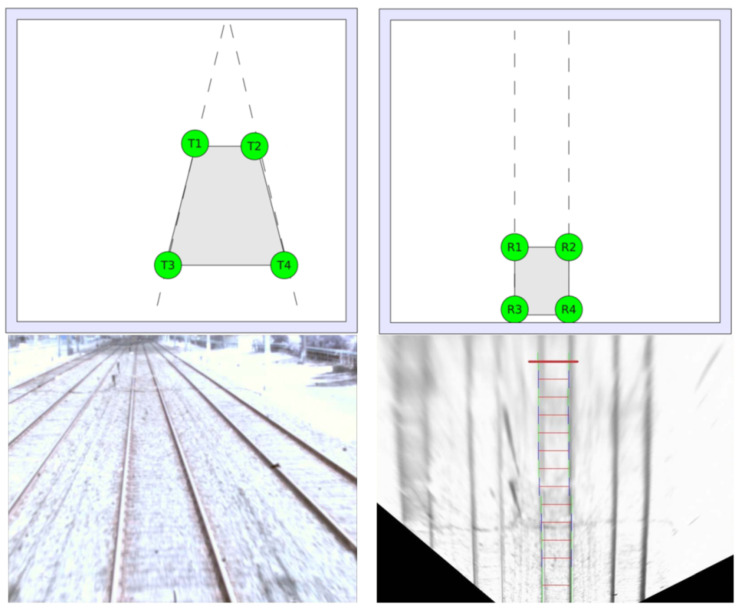
Using of rail tracks geometry for the purpose of rail tracks detection. Projective transformation of the on-board camera image (**left**) into a bird’s-eye view image of the rail tracks (**right**), extracted from [33].

The vast majority of algorithms in this category are geometry based. Starting from the rail track geometry as consisting of two parallel lines, several published works exploit methods for lane detection, which is a fundamental yet challenging task in autonomous driving and intelligent road traffic systems. Those works extend the line detection methods using the geometric characteristics specific to rail tracks. For example, the method proposed in [31] is based on the fact that the distance between the rails and other geometric details of the railway are known a priori for each country. In particular, the presented method uses the rail points that are also on a normal to one of the rails as the base markers for determining spatial coordinates as part of a Perspective-2-Point solution. The method for rail detection presented in [32] is based on three hypotheses: the gap between the rails at the initialization stage is constant, in the bird’s-eye view, the rails are parallel, and, in the projectively distorted image, the rail tracks intersect in a vanishing point. The author in [19] presents an approach for recursive estimation of the edges of the rails in the on-board monofocal camera pictures, which are then tracked from bottom upwards by means of a discrete Kalman filter. The geometry of rail tracks is exploited also in [33] in a method for rail tracks detection for determining the obstacle-free range for rail track maintenance vehicles. The presented method is based on projective transformation of the camera image into an image where the rails are parallel, and on tracking the two rail curves simultaneously by evaluating small parallel segments. The idea behind the projective transformation-based method is to get the so-called bird’s-eye view of the rail tracks that is less distorted by perspective than an observer’s view in the driver cabin of the train (Figure 1). Besides the parallel rail tracks, the bird’s-eye view also enables exploitation of the fact that the sleepers between the parallel rails are spaced regularly.

Some of the published papers present methods that are based on the extraction of specific rail track characteristics from the camera image—for example, their sharp edges that provide a large image gradient. For example, the authors in [21] present a railway tracks detection and turnouts recognition method that is based on extracting an image feature known as the Histogram of Oriented Gradients (HOG). First, HOG features are computed and the integral image is generated. After this, the railway tracks are extracted from the integral image using a region-growing algorithm. The method is based on the fact that the variance of the HOG features corresponding to the image blocks that contain rail track is relatively large since such a block always contains prominent edges. Evaluation results showed that the method proposed in [21] was superior to other related methods [23,27,34]. In [23], gradient-based edge detection is used for rail tracks extraction. Extracted rail tracks are then tracked for the purpose of detecting rail switches. The authors in [27] present a method that detects the rails by matching image edge features to candidate rail patterns, which are modeled as sequences of parabola segments. For rail areas near the camera (near-range rails), patterns are pre-computed while, for far-range rail areas, they are generated on the fly. An approach representing the advancement of [27] is presented in [26]. The improvements include a semi-automatic method for generating rail patterns for the near-range rails and multiple extractors for increasing robustness in the far-range. Methods are investigated for making rail extraction faster and more robust by exploiting the fact that the rails should have similar position and curvature in consecutive frames. In [34], an algorithm based on dynamic programming is used to extract the train trajectory and the area of rail tracks in front of the train. First, the railway features, the rail tracks vanishing point, and the distance between the tracks are extracted from an input image using gradient-based edge detection. Feature extraction is followed by a Hough transformation (a widely used CV method for detection of simple shapes such as straight lines) for line detection. Then, dynamic programming is applied to compute the path which gives the minimum cost to extract the railroad track area. The proposed algorithm also uses dynamic programming to extract the left and right rails simultaneously and does not need any static calibration process. The Hough transformation is also used in [35], however not on the original input on-board camera image, but instead on a transformed bird’s view image in which the parallel characteristics of rail tracks are maintained and also dimension information such as the gauge width between two rail tracks is known. The bird’s view image is obtained using Inverse Projective Mapping (IPM). Then, the IPM image is segmented using modified edge-based image segmentation, and the Hough transformation is applied to the edge map in order to extract the rail track through detection of the line segments. The detected line segments are combined with some geometric constraints from a priori knowledge leading to robust extraction of the rail tracks. The continuation of work [35] is presented in [36], where a method for rails extraction by matching the edges extracted from the real IPM image with the candidate parametrically modeled rail patterns is introduced. In addition, for the generation of rail patterns, the geometric constraints of the rails are taken into consideration. A distance transform is used as the similarity measurement between the candidate pattern and the extracted edge map with the same size as the pattern. An IPM method was also used also [37]. The presented method actually uses lane detection techniques from car driver assistance systems to assist in rail tracks detection for autonomous trains and also uses the geometric constraints of the rails. The created IPM image is filtered using a Difference of Gaussian (DoG) smoothing filter with a large kernel size to eliminate motion blur and camera focus error. This DoG filtered image is segmented using global thresholding to create a binary image which is then split vertically into 10 equally sized slices. This breaks up longer lines and allows extraction of parallel line segments. The authors in [38] present a robust approach that partitions a video frame into four sub-regions: near, far, remote and horizon. Each sub-region is filtered by two-dimensional Gabor wavelets at different scales. Considering the size and the orientation range of the rails at each partition, the responses of the Gabor wavelets are tuned to specific frequencies to enable rail edge highlighting together with noise filtering. The four sub-regions have different characteristics. Firstly, the horizon sub-region does not contain any information about the railway tracks, so it can be ignored. In close to remote sub-regions, the visual appearance of the thickness of the rails decreases while their directionality (i.e., orientation spectrum) increases. Being a method in the frequency domain, the proposed method for automatic extraction of the railways is relatively insensitive to fine-tuning of the processing parameters, which is a typical problem of methods based on image processing methods in the (image) spatial domain.

In contrast to the studies mentioned above that use presumed knowledge and assumptions about the geometry of railway tracks, in [39], a statistical analysis of railway track is provided. The analysis relies on various video recordings to quantify and bound variation. Furthermore, based on the results of these statistical analyses, this paper introduces a method to predict the railway tracks by means of polynomial approximation, which is followed by multi-layer perceptron networks.

The performance of all of the traditional CV methods for rail track extraction mentioned above may deteriorate due to varying lighting conditions, change in weather conditions, and complex backgrounds. Traditional methods that use line or edge features to detect rail tracks may have a good performance in a fixed scene, but their performance may deteriorate fast as the scene changes. As a train travels along the track, the background is constantly changing, and the “hand-crafted” features of traditional CV methods may not be robust enough to meet the requirements. In addition, the environment of the moving locomotive can present specific challenges for an installed on-board camera—for example, significant vibrations can cause blurring. In order to cope with these real-world problems, in [40], the so-called Structure from Motion (SfM) pipeline was specifically designed to support railway infrastructure monitoring systems using a monocular on-board camera.

#### 2.1.2. Detection of Obstacles on the Rail Tracks

**Figure 2:**
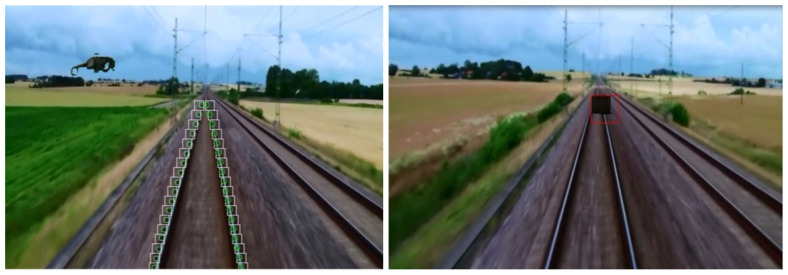
Detection of objects in the ROI—region of detected rail tracks. (**left**) Windows-based systematic search for objects along the detected rail tracks ([17,41]); (**right**) digitally added stationary object and its detection ([17,41]).

Once the rails are detected in the on-board camera image, the obstacle detection problem can be reduced to a simpler domain, where only the image region within the predefined margins around the rails needs to be analyzed. Most published traditional CV-based obstacle detection methods are based on searching along the detected rail tracks or on searching within the image ROI defined upon rail tracks detection. In the method presented in [29], once the image ROI is extracted through rail extraction, different detection methods are applied for moving and stationary objects. An optical flow method is applied for moving obstacle detection within the ROI while stationary objects are detected using the so-called Sobel edge detection method followed by morphological processing of the edge detected image. The method presented in [41] deals with rail track identification also using the Hough transform, followed by obstacle detection using CV algorithms. For the obstacle detection stage, the image is first filtered using the Canny edge-detection algorithm and then the edge contours in the image are extracted. Small and disconnected contours are then eliminated. After filling the remained contours, a systematic objects search is started using the rails as guide. The algorithm was evaluated on images with digitally added obstacles. The continuation of work [41] is presented in [17] where the Hough transformation for detecting the rails was again used. Once the rail tracks are detected, on each rail a systematic search is done (from bottom upwards) to detect hazardous obstacles (Figure 2 left). This method was effective for detecting fixed objects in front of the train and also obstacles close to the rails (Figure 2 right). The paper also presents a different method for detecting dynamic obstacles, based on the concept of optical flow between frames. The method identifies candidate hazardous objects and ignores irrelevant background moving elements. The trajectories of the candidate hazardous objects are tracked and used to predict whether their future trajectories could lead to a collision with the train. Bottom-up adaptive windows are also used to find obstacles along detected rails in [32]. After determining the gradient image, the start of the rails is located in order to create bottom-up windows and to check for obstacles. However, the authors claim that cases of obstacles on railways are rarely filmed or photographed, and, consequently, it was not possible to evaluate the method on real-world images. Instead, the test videos of rail track scenes were modified by adding digital obstacles of different nature, shape and obstruction trajectory.

**Figure 3:**
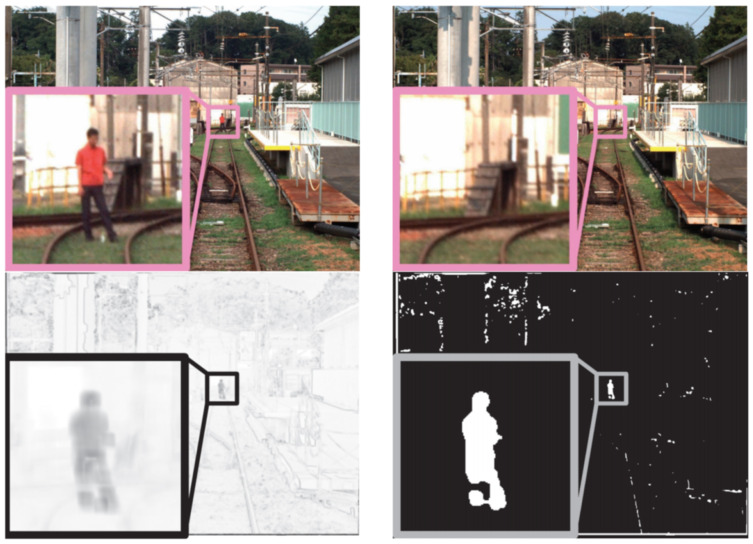
Detection of objects using background subtraction (extracted from [42]). (**Top left**) Current frame with an object; (**Top right**) reference frame without object; (**Bottom left**) difference in color between current and reference frame; (**Bottom right**) detected object using segmentation of difference image.

In [42], a method for obstacle detection based on so-called background subtraction that is applicable to moving cameras and that uses reference images as baselines is presented (Figure 3). In this paper, background subtraction was realized by comparing the live (current) on-board camera image of the scene in front of the train with a reference image. The presented method first calculates frame-by-frame correspondences between the current image and previous camera image sequences captured at the most similar location to the actual camera image sequence, and it finds a reference frame using image sequence matching. Then, obstacles are identified by applying image subtraction of the current frame from the corresponding reference frame, assuming that there are no obstacles in the reference frame to create a ‘difference frame.’ As a result of the similarity of the current and reference frames, only the image areas with obstacles in them in the current frame should have pixels values that differ significantly from zero in the difference frame. The method presented in [22] is also based on background subtraction that is comparing the live images from on-board cameras with images captured by cameras mounted on other trains operating earlier at the same route. The authors in [43] present a new approach for detection of dynamic objects in railway scenes in front of the train, which uses computer vision and Principal Components Analysis (PCA). For this, on-board camera images of the railway static environment are first captured to calculate the transformation matrix used by PCA. By means of this transformation matrix, the consecutive camera images are projected into the transformation space and recovered later using inverse transformation. Motion detection is accomplished by calculation of the Euclidean distances between the original and recovered images. Image regions whose Euclidean distances are larger than a pre-defined threshold value are considered to belong to detected dynamic objects.

The majority of published papers, including the above-mentioned ones, propose obstacle detection methods that detect arbitrary objects (anomalies) on the rail track without consideration of the particular object class. In contrast, the authors in [6] explicitly consider ‘pedestrians’ and ‘vehicles’ as object classes, and suggests the use in railway systems of established methods for pedestrian detection and vehicle detection from road vehicle systems.

#### 2.1.3. Obstacle Distance Estimation

In order to improve railway safety through autonomous on-board OD, obstacle distance estimation has to be an integral part of OD. Namely, the only way for a train to avoid a collision with an obstacle on the rail track is to come to a complete stop before making contact with it. In other words, collision avoidance is only possible if the detection distance exceeds the train’s stopping distance. The exact train stopping distance depends on many factors including the mass distribution of the train, the speed of the train when the brakes are applied, the deceleration rate achievable with maximum brake application, brake delay time, and the gradient of the track [44]. For example, according to national regulations in most EU countries, the stopping distance of a freight train pulling 2000 t of cargo at 80 km/h is approximately 700 m. This long-range stopping distance represents a specific challenge for trains when compared to road vehicles, where a detection distance of up to 200 m is sufficient. Even though the estimation of distances between detected objects and on-board vision sensors (distances between obstacles and the train) is crucial for obstacle detection to react early enough, the majorities of published CV works do not consider distance estimation at all, or consider it implicitly through detection of the obstacle-free ROI along the rail tracks only. Although the need for long-range obstacle detection is well established, the published works considering obstacle distance estimation consider relatively short-range distances as discussed in the following. The authors in [29] presented a method of detecting obstacles which monitors the status of the track for 600 m or over ahead of a train, using an image sequence taken by an ultra telephoto lens camera mounted on the train. However, there are no details provided on estimation of distances to individual objects or to maximum monitoring distance range. For the purpose of enabling automatic docking of a locomotive with a train, the authors in [31] considered an algorithm that inferred the distance from the locomotive to the first wagon of the train by reformulating this distance as a the solution of a Perspective-2-Point problem. Because of the application, the considered distances are short-range. Evaluation results showed that, on a straight section of railway track at distances up to 50 m, an absolute error of measuring the distance to a locomotive or wagon did not exceed 1.2 m. The problem of calculating the distance between a shunting locomotive and a wagon using video from a camera mounted on the locomotive is also considered in [45]. Here, in a two-stage method, first, the rail tracks were detected using traditional CV methods, Canny edge detection and Hough line transform; then, detection of Haar features and a Neural Networks-based search for wagons in the detected rail track region was performed. If no wagons were detected in images covering the shunting locomotive visibility range of 100 m, then the system issued a signal that the rail tracks were not occupied. In addition, the image coordinates of the farthest detected point of the obstacle-free rail tracks were calculated, and, based on these coordinates, the distance to that point was calculated. The method assumed no other objects on the rail tracks apart from the wagons. Instead of estimating distance to an individual object, the method proposed in [33] detected reliably the obstacle-free zone to more than 100 m. Detection of such an obstacle-free zone is sufficient for the considered application, an anti-collision system for rail track maintenance vehicles that monitors the space ahead for possible obstacles with a particular objective of maintaining a safe distance between maintenance trains. Methods with the potential for detecting the railroad track space at a long distance and rails at longer distance are presented in [26,34], respectively; however, neither paper explicitly considered distance estimation.

Some systems which were primarily based on vision sensors used additional range sensors to support distance estimation. For example, the authors in [8] present a system where a laser rangefinder is combined with a camera for distance estimation. In the described system, if the distance to an obstacle is critical in relation to the sum of the braking distance, idle running distance, and a ‘safety margin’ distance (total distance 1 km), an alarm is issued to the driver. Ref [16] presented a multi-sensor system consisting of look-ahead sensors, video cameras (optical passive sensors), and Lidar (optical active sensor) having a look ahead range of up to 400 m under typical operating conditions. The prototype system was assessed on a test vehicle named “Train Control TestCar” driving at speed of up to 120 km/h over long distances across Germany. One of the specifications for the presented system was that objects at a position ahead of the vehicle that will be reached in 10 s time must be detected with “high probability.” For example, if the vehicle travels at 100 km/h, an obstacle must be detected with “high probability” at 300 m distance. However, the train braking distance was not explicitly considered. In [11], results of tests of a system for small test objects of 0.4 m${}^{2}$ in size up to a distance of 250 m were published. A key result was that persons wearing a warning waist coat moving on the track were detected at distances up to 300 m. The main sensors of this system were three progressive scan CCD video cameras. Two of these cameras were used in a stereo setup with a 0.35 m baseline and were fitted with 12 mm focal length lenses for monitoring the near range up to a distance of approximately 50 m. The third camera with a 35 mm lens was used for the far range. Additionally, a sensor from an automotive adaptive highway cruise control system was used, a multi-beam infrared radar with a detection range of 150 m and an opening angle of 8 degrees. This was mounted on the front of the train at a height of 0.6 m.

In [7], an on-board multi-sensor obstacle recognition system for regional trains is presented that satisfies the following requirements (quoted directly here): (1) detect all relevant obstacles to assure safe operation inside the railway track clearance in a distance of less than 80 m within direction of movement; (2) any object larger than 40 × 40 cm is to be considered as a relevant obstacle; (3) the assumed emergency braking (deceleration) performance is $2.73\;\frac{m}{s^{2}}$, matching German electric tramway regulations. The 80 m distance corresponds to particular speed restrictions for the regional railway operations considered. The same maximal distance range of 80 m was considered in [12]; here, a system was presented that satisfied the requirement of detection of obstacles inside a track’s clearance volume of at least 0.3 m × 0.3 m × 0.3 m at a distance between 10 m and 80 m ahead of the train. It was concluded that the system performance depended heavily on the accuracy of the input track information, so, when it was inaccurate, the system could fail in certain situations.

### 2.2. AI-Based Methods

In recent years, the advancement in neural network technology has enabled great improvement in object detection based on AI in road traffic applications, but research regarding rail transport has lagged behind. However, in the last few years, there has been a growing interest in AI-based applications to railway systems in order to avoid use of “hand-crafted” features such as those used in traditional CV methods and instead to adaptively extract image features through Convolutional Neural Network (CNN) structures. The results already achieved in other transport sectors, mainly automotive, have supported the development of AI in railways. However, in contrast to the automotive field, where large scale image datasets such as KITTI [46] are available for efficient evolution of effective object detection models using road scene images, the railways field has, to the best of our knowledge, only one relevant dataset and this is limited in scope. This dataset, RailSem19, introduced in [47], is the first publicly available dataset specifically for semantic railway scene understanding. RailSem19 contains images acquired from the point of view of a train and a tram, and it contains specific annotations collected for a variety of tasks, including the classification of trains, switch plates, buffer stops and other objects typically found in railway scenarios, but not anomalies and obstacles. In the absence of relevant public datasets, the majority of AI-based methods for rail tracks detections and obstacle detection in railways are based on custom made and non-public datasets as reviewed in the following.

#### 2.2.1. Rail Track Detection

AI-based rail tracks detection methods reformulate railway detection tasks from railway line detection or edge detection problems, as considered in traditional CV methods, to image segmentation problems. In [48], an efficient rail area detection method based on Convolutional Neural Network (CNN) was presented. The method consists of two parts, extraction of the rail area and its further refinement. The latter consists of maximizing the matching of the contour of the extracted rail area using a polygon fitting method, and thus getting a more accurate outline of the rail region. The presented CNN achieved rail area extraction at the pixel level of resolution. The used network was inspired by the commonly used segmentation network SegNet [49], an encoder–decoder architecture for image segmentation, which trades off detection precision and extraction efficiency and consists of an encoder to extract the features of the rail area in the image, and a decoder which upsamples the encoder’s feature maps to match the input resolution and also classifies the rail track area in the image in a ‘softmax’ layer. The training and testing of the proposed network was done using a bespoke dataset named BH-rail, which was annotated according to Cityscape dataset standards [50], i.e., pixel level labeling for the rail region and the non-rail region was used. The dataset was recorded on the Beijing Metro Yanfang line and the Shanghai metro line 6 with an on-board RGB camera. The BH-rail dataset has 5617 annotated images and covers representative real scenarios including tunnel and elevated environments.

**Figure 4:**
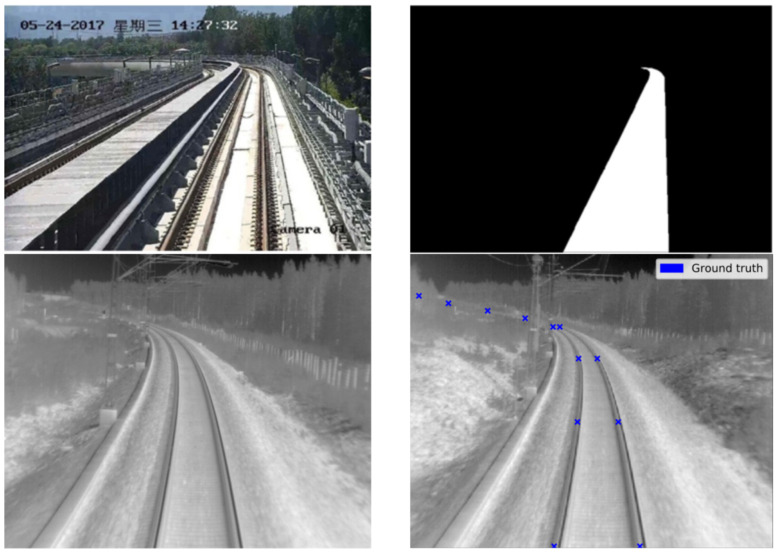
Image annotations for AI-based rail tracks detection. (**Top**) Pixel level annotation for instance segmentation: original RGB image left, annotated image right ([18]); (**Bottom**) key points annotation for rail track key points detection: original Thermal image left, annotated image right ([53]).

In [18], a DL-based segmentation algorithm for railways named RailNet is presented. RailNet is an end-to-end DL-based railway track segmentation algorithm that consists of a feature extraction network and a segmentation network. The feature extraction network uses a pyramid structure to propagate features from top to bottom to obtain a hybrid feature vector. The segmentation network is a convolutional network for generating the segmentation map of the railway. In order to train and test the RailNet network, the authors created the Railroad Segmentation Dataset (RSDS) that includes 3000 images, 2500 images for training, 200 images for validation and 300 images for testing. All of the images came from a real-world train’s driving environment. In the ground truth labelling of the railway datasets, the railway was defined as all pixels between two rail tracks (Figure 4 Top). During training, the authors also applied a data augmentation algorithm to enhance training samples, but no details of this algorithm were given. The weights of the RailNet backbone neural network (which is ResNet50 [51]) were initialized using weights trained by the publicly available large-scale ImageNet Dataset [52].

A rail detector based on an on-board thermal camera for detecting rails at long range and, in night conditions using machine learning and in particular using DL and a CNN, is presented in [53]. The developed CNN, a modification of a CNN already proven in other image classification applications, detected key points along the rails and put the largest weight on the area furthest away from the train. A labelled dataset for CNN training and testing was produced manually (Figure 4 Bottom). The data were recorded by an on-board thermal camera mounted on the top of the locomotive. Recording was done during different times of the day, and four video sequences were used in the dataset. All the frames in these sequences were labelled, except for the frames containing multiple railway lines. The performance of CNN-based method for detecting key features on the rails was compared with the performance of a previous system from the same research group, which was based on a traditional CV method [24]. The trial results demonstrated the capability of CNN-based methods for automatically learning rail features, in particular distant features that could potentially be used in place of “hand-crafted” features.

#### 2.2.2. Obstacle Detection

Published AI-based methods for obstacle detection in railways have mainly used DL-based methods/networks for object detection already proven in other applications. As presented in the following, for the purpose of object detection in images of railway scenes, these established DL-based networks are used in their original form, as trained with large publicly available object detection datasets, or they are re-trained with custom made railway datasets.

**Figure 5:**
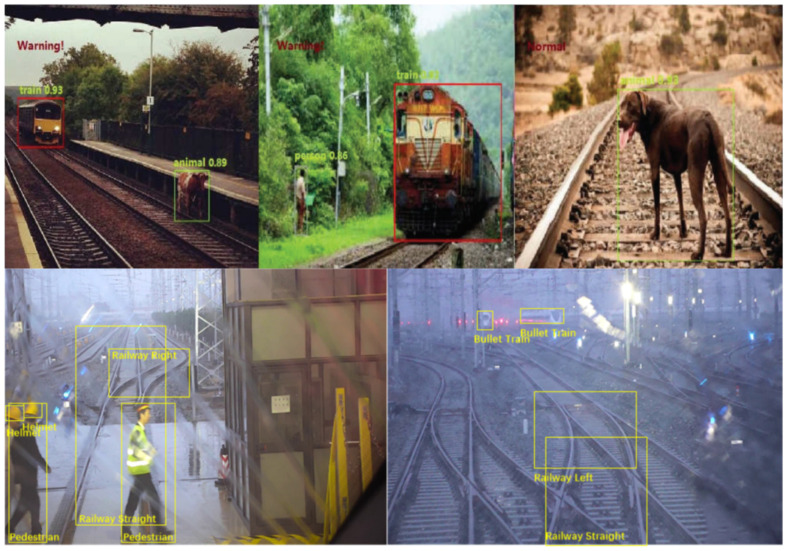
Custom made railway datasets for AI-based obstacle detection. (**Top**) Example images from a dataset generated using images that are publicly available on the Internet (extracted from [54]); (**Bottom**) example images from a dataset generated using real-world images from shunting environment (extracted from [13]).

In [54], a DL-based method for detection of obstacles on rail tracks is presented that transforms the obstacle detection problem into a target object detection problem. A CNN based feature extractor to detect objects in railway images is proposed which is based on the Faster Region based Convolutional Neural Network (Faster R-CNN, [55]) framework. A dataset of 20,000 images of outdoor railway scenes, which are publicly available on the Internet, was used for obstacle detection network training (Figure 5 Top). The objects found in the images were divided into three categories: train, people and animals. The training set consisted of 15,000 images and the test set consisted of 5000 images. The Faster R-CNN was also used in [56] for identification of objects on the rail tracks. The method presented can be considered as a hybrid approach where traditional CV is used for rail tracks detection and the AI-based method is used for the identification of objects on the detected rail tracks in thermal camera images. In the first stage of processing, the traditional CV techniques (Canny edge detection and the Hough transform) are used for rail tracks (ROI) detection. For detecting objects on the rail tracks, input thermal camera images are first transformed to HSV color space images to remove the noise, where the less bright objects in these images (unwanted surroundings such as trees and buildings) are considered as noise. In this way, the objects of interest (possible obstacles on the rail tracks) are in effect restricted to living creatures on the rail tracks, as these appear as brighter than their surroundings in thermal images. After noise removal, Faster R-CNN is applied to identify the objects from the HSV segmented frames. The implemented Fast R-CNN consisted of two modules; the first is a deep fully convolutional network used to propose regions, and the second is the Fast R-CNN detector itself that identifies the objects using region proposals. The thermal camera was mounted on the front top of a moving train. For the proposed work, the camera recorded video at a resolution of 360 × 450 pixels. The authors stated that 749 frames were used for evaluation of the proposed object detection method, but do not detail whether the recorded thermal camera images were used for re-training of the used network, or the pre-trained network was just applied to the recorded thermal camera frames.

The Railway Object Dataset built by the authors of [13] was used for the development and evaluation of a method that can detect seven different types of objects typically present in a railway shunting environment: bullet train, railway straight, railway left, railway right, pedestrian, helmet and spanner (Figure 5 Bottom). The method is based on CNN and is known as a Feature Fusion Refine neural Network (FR-Net). It consists of three connected modules: the ‘depth-wise-pointwise’ convolution module, which improves detection in real time; the ‘coarse detection module,’ which approximates the locations and sizes of prior anchors to provide better initialization for the subsequent module and also reduces the search space for the classification; and the ‘object detection module’ that provides more accurate object locations and predicts the class labels for the prior anchors. An improvement to the FR-Net method is proposed in [14], an object-detection method that uses the so-called DFF-Net (Differential Feature Fusion CNN). DFF-Net is an end-to-end object detection network with a fully convolutional network architecture. DFF-Net includes two modules: the prior object detection module, which produces initial anchor boxes; and the object-detection module, which applies a differential feature fusion sub-module onto the initial anchor boxes to enrich the semantic information for object detection, enhancing the detection performance, particularly for small objects. The Railway Object Dataset used for training and testing the methods proposed in [13,14] consisted of 7342 images of railway shunting scenes recorded in different lighting and weather conditions. A DL-based method for detecting small objects in railway traffic scenes is proposed in [25], which also uses this Railway Object Dataset for training and testing. The proposed method is based on the FE-SSD (Feature-Enhanced Single-Shot Detector) which exploits the capabilities of a prior module detection mechanism named RON–Reverse connection with Objectness prior Networks [57], and Faster Better Network (FB-Net, [58]); it does this by fusing the adjacent feature maps to learn deeper semantic information and by using a feature transfer block. The evaluation results showed that the proposed method provided a good balance between accuracy and real-time performance for object detection and significantly improved the small-object detection performance with respect to several other considered methods derived from same original DL-method.

A multi-stage obstacle detection method is also proposed in [59]. The method has two steps: feature map creation and feature fusion. In the first step, the input image is converted into multi-scale feature maps using a Residual Neural Network (RNN). Multi-scale features maps improve the identification of objects of different sizes at different distances. Specifically, low-level features lack semantic information but provide precise object location information, while high-level features are rich in semantic information but provide only approximate location information. Once the multi-scale features maps are created, a series of convolution layers are added to extract features, and the network calculates a confidence score and bounding boxes for possible obstacles. For training and testing the network, the authors made a custom dataset which contained large-scale urban rail transit video frames taken by an on-board HD camera. The dataset was gathered from three metro railways in China with camera image resolution of 1280 × 720 pixels, and has 6384 images with 5107 images for training and 1277 for testing. The dataset covers different lighting and weather conditions and its images have three classes of manually annotated objects: train, luggage, and humans.

In publications [60,61], a novel machine-learning based method named DisNet was presented which works with on-board cameras to detect possible obstacles in front of the train. DisNet consists of two parts: the first part performs DL-based object detection, and the second part is a multi-hidden layers neural network-based system for distance estimation. An object detector can be any bounding box-based DL-based method which extracts the bounding box of an object detected in the input image as well as the object class. The results presented in [60,61] were obtained using one of the state-of-the-art one stage DL-based methods for the prediction of detected objects bounding boxes named YOLOv3 [62]. The main advantage of YOLO is its speed, making it appropriate for real-time applications such as on-board OD in railways. The distance estimator is a feedforward artificial neural network that consists of three hidden layers, each containing 100 hidden units. DisNet estimates the distance between each detected object and the on-board camera based on the features of the object Bounding Box (BB) extracted by the YOLO-based object detector. The YOLO model used was first trained with the Microsoft COCO dataset [63] of images of everyday scenes containing common objects in their natural context, consisting of 328,000 images of 80 easily recognizable object classes. In total, 2.5 million objects are labelled in the images of the dataset, and about 3500 images of railway scenes are labelled with the object class “train.” However, the COCO dataset does not contain images of explicit railway scenes of objects on the rail tracks and, moreover, it does not contain images of distant objects. In order to enable the YOLO model to detect objects in railway scenes, with particular focus on long-range distance objects, the starting YOLO model was re-trained on the custom long-range railway dataset developed as part of project SMART [61]. In total, 998 images captured with SMART RGB cameras were used, with 2238 labelled objects of classes human, car, bicycle and large animal. These images were recorded by a multi-camera system mounted on a Serbia Cargo locomotive, during operational runs in different illumination conditions along a 120 km long Serbian section of the pan-European ‘Corridor X’ route.

#### 2.2.3. Distance Estimation

Although papers describing AI-based methods for OD in railways often state that distance estimation between the on-board camera and detected objects ahead of the train is an important element of OD, such papers rarely include details of distance estimation even if this is an implied or mentioned feature of the presented method. For example, there are several works published on obstacle detection in shunting applications where short–range object (wagon) detection is assumed. In [14], it is stated that, in addition to the on-board camera that captures images, the system also has millimeter-wave radar for measuring distances between the detected object and train, but no details are provided on the range of distances measured or how the measured distances are further used. The system proposed in [25] is also a short-range system mainly aimed at object detection in shunting mode with train speeds less than 45 km/h. Another obstacle detection system for shunting locomotives is presented in [64], this one being a stereo-vision and CNN based system for determining the safe distance between the locomotive and the wagon coupling device. However, no details are given about distance estimation of the objects detected.

Another group of papers present the use of long-range cameras for viewing of the rail tracks in long-range and, by implication, for distance estimation of distant objects within this viewed long-range. For example, in the work presented in [56], an on-board thermal camera was used which had a distance range of up to 1500 m. The paper presents results of object detection within the camera visibility range, on the rail tracks’ portions visible in the camera image. However, no details are given on the estimation of distances to individual detected objects. A different on-board thermal camera-based system for finding rails at long-range is presented in [53]. However, no details on the detection distance range are given.

**Figure 6:**
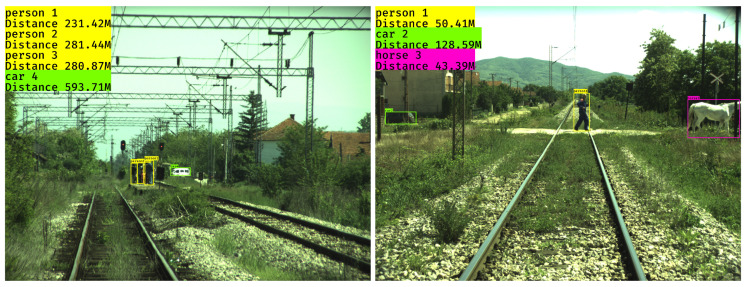
DisNet estimation of distances to objects in a rail track scene from the RGB camera image (extracted from [61]).

The only known published work explicitly describing obstacle distance estimation is presented in [60,61]. The main part of the on-board vision-based obstacle detection system presented in [60,61] is the distance estimator named DisNet. DisNet estimates the distance between each detected object in the camera images and the on-board camera, using the features of the object Bounding Box (BB) extracted by the DL-based object detector. In other words, DisNet learns the relationship between the size of the object BB and the distance of the object to the on-board camera. The evaluation tests performed in an operational railway environment demonstrated that this integrated on-board vision-based obstacle detection system fulfilled the functional requirements of mid- and long-range obstacle detection. This system extended the state-of-the-art by providing long-range object detection and identification of obstacles in the mid-range (from 80 m up to beyond 200 m) and in the long-range (up to 1000 m). Some of the DisNet results on distance estimation of real-world objects are given in Figure 6.

## 3. Evaluation Tests

The papers published so far on OD in railways can be put into three categories based on the source of the data used for the evaluation, the first being data publicly available on the Internet, the second being custom made datasets, and the third being data from real-world field trials.

Due to the limited availability of real world video footage in which obstacles appear in front of trains, the method presented in [41] artificially added obstacles to route recordings published on the Internet. The system was tested using these modified videos, and a particular modified video of the route between Landskrona and Ramlösa in Sweden was used for evaluating the system performance. A total of 31 digital obstacles of different nature, shape and obstruction trajectories were added to the test video. The evaluation metrics were “number of true positive object detections (TP),” “number of false positives (FP)” and “number of false negatives (FN)“. The presented method achieved high detection rate of TP equal to 30. The same evaluation metrics were used in [66] for the evaluation of the proposed conventional (CV-based) human detection method which exploited the temporal continuity between frames by using a combination of the motion vectors obtained by optical flow estimation and the thresholding of the similarity of humans detected in subsequent frames. The method was evaluated on a train driver’s view video available on the internet, recorded while a train was approaching a platform. The test video sequence was of limited length, containing only 12 frames with a resolution of 1920 × 1080 pixels. Instead of using the absolute values of TP and FN, the evaluation metrics were expressed through the percentage of humans that the method failed to detect (FN) and percentage of humans the method correctly detected (TP, also named “Detection”). As discussed in Section 2.2.2, the method proposed in [54] was tested on 5000 internet sourced images of railway scenes with unexpected objects (people, animals, trains) on the rail tracks. The main evaluation metric used for obstacle detection was mean Average Precision (mAP). The detection performance of the proposed method was evaluated by comparison of the mAP, achieved on the test set, with the mAP of two widely used CNN-based feature extractors. The evaluation showed that the proposed method had better performance. In addition, the performance of the system was evaluated by the accuracy of issuing the alarm after detecting hazardous objects, which reached 94.85%.

AI-based methods are usually evaluated on the images in custom constructed datasets. For example, the CNN-based method for rail tracks detection proposed in [48] was evaluated on the custom dataset recorded in real-world conditions on the Beijing metro Yanfang line and Shanghai metro line 6 with an on-board camera image resolution of 1280 × 720. The generated rail-dataset of 5617 annotated images includes representative real scenarios such as rail tracks in tunnels and in elevated environments. The annotated dataset was split up into a training set and a test set with 4494 and 1123 images, respectively. The accuracy of the proposed method was evaluated using the commonly adopted Mean Intersection-over-Union (MIoU) metric and the mean pixel accuracy (MPA) metric. Each of these metrics is based on the calculation of the number of pixels of class *i* detected to belong to class *i* (TP), the number of pixels of class *j* detected to belong to class *i* (FP) and the number of pixels of class *i* detected to belong to class *j* (FN). The evaluation results showed that the proposed method achieved high accuracies, 98.46% MIoU and 99.15% MPA, on the used custom dataset. The DL-based method for railroad area detection proposed in [18] was evaluated on the custom railroad datasets called the Railroad Segmentation Dataset (RSDS) that included 3000 images: 2500 images for training, 200 images for validation, and 300 images for testing. The images of RSDS were recorded in a real-world environment—autonomous trains with a running speed less than 60 km/h—with an on-board camera of resolution 1920 × 1080 pixels. To evaluate the performance of the proposed method, the authors used typical metrics for semantic segmentation evaluation such as pixel accuracy (PA) and mean Intersection over Union (MIoU). In addition, since the railroad detection was based on pixels, it could be considered as a classification task, so the authors also used evaluation metrics typical for image classification: precision (ratio of true positive detections TP and the total number of detections, including true as well as false positives, TP + FP), recall (ratio of true positive detections TP and the total number of positive instances in the dataset TP + FN), and F-score as the trade-off between precision and recall. The evaluation results indicated a very good performance of the proposed method with 89.8% MIoU, 91.6% PA and 86.8% F-score while achieving 20 frames per second processing speed. With these achieved results, the proposed method outperformed four state-of-the-art methods for scene segmentation that were tested on RSDS to compare their performance with the proposed method. The methods proposed in [13,14] were evaluated on the images of the custom made Railway Object Dataset for object detection in a shunting mode environment; to ensure diversity in the data, the data were recorded under different lighting and weather conditions. In addition, 7342 sample images with a resolution of 640 × 512 pixels were recorded using on-board cameras. The dataset images were labelled manually and seven different classes of objects usually present in railway shunting environment were annotated: bullet train, railway straight, railway left, railway right, pedestrian, helmet, and spanner. The last two object classes are objects that may be left behind on the rail tracks by railway workers. In experiments conducted on a railway traffic dataset, both proposed methods were compared with four state-of-the-art detectors. The method presented in [13] achieved a mAP of 90.12%, which was far better than those of the other considered detection methods. The method presented in [14] achieved 89.53% mAP, suggesting that the presented method made a good trade-off between effectiveness and real-time performance. The same Railway Object Dataset extended with approximately 1400 further images of the same shunting scenes was used for the evaluation of method proposed in [25].The experimental results indicated that the proposed approach was superior to other methods derived from same original DL-method, particularly for small-object detection. It achieved a mAP of 89.5% at a frame rate of 38 frames per second. The achieved frame rate indicated that the proposed method could be used for real-world railway OD. The CNN-based method proposed in [59] was evaluated using a custom dataset which contained large-scale urban rail transit video frames taken by on-board HD cameras. The dataset of 6384 images was gathered from three rapid transit and metro lines in Hong Kong, Beijing and Shanghai and was divided into two groups: 5107 images for training and 1277 for testing. The dataset contains images in daytime and night, sunny and rainy days. Average Precision (AP) was used to measure the accuracy of the proposed object recognition method in each considered object class: train, pedestrians and luggage. In train detection, the method achieved 95.38% AP, which was 12% higher than the AP of an established state-of-the-art method. In terms of small obstacles (pedestrians and luggage), the APs were smaller, respectively 90.49% and 90.38%; however, the achieved evaluation results demonstrated that the proposed method still performed well. Besides APs per object class, the average of all individual APs, mAP, was calculated to serve as an aggregate indicator of the performance of the proposed CNN on the dataset used. The achieved mAP was 91.61%, which was much higher than the mAP calculated for three state-of-the-art related methods applied to the same dataset. The CNN-based method for detection of key points on the rail tracks presented in [53] was evaluated on 2800 random frames captured by a thermal camera of resolution 640 × 512 pixels. The camera was mounted on the top of a running train and was placed in custom housing protecting the camera lenses, and the data were collected in northern Sweden in April 2015 during different times of the day. Four video sequences were used to build the dataset for training and testing of the proposed CNN-based method. Eight different approaches were used when training the proposed CNN, and each one resulted in a different rail detector. To evaluate whether rail detected by each detector was good or not, a tailored loss function was used as the evaluation metric. In addition, the performance of the proposed CNN-based method was compared with the performance of a previous system from the same research group, which was based on a traditional CV method [24]. The trial results demonstrated the capability of the CNN-based method which, by its nature, automatically learns rail features including distant features to be be used in place of the “hand-crafted” features of traditional CV-based method.

Real-world field evaluation trials were described in [16]. A public rail track near Munich was used as the test track. The test vehicle was driven towards wooden plates painted to simulate typical obstacles with different attributes which were positioned on and beside both straight and curved (500 m radius) track. The sizes of the plates were 0.4 m${}^{2}$ (simulating small objects like children) and 2 m${}^{2}$ (simulating big objects like passenger cars). The ability of the system to detect people wearing reflective safety jackets was also investigated. As the locomotive approached each obstacle, at a particular distance, it was recorded that the obstacle’s distance had been reliably estimated for the first time. This estimated distance gave useful data points about the system’s performance even though the number of samples was very small (19 samples for small obstacles and 7 samples for big obstacles). The performance of the system was evaluated by its comparison with human driver performance; it was concluded that, for large distances, the human driver’s detection capabilities were better.

The obstacle detection and distance estimation system presented in [60,61], which consisted of DL-based object detection followed by multi-hidden layers neural network-based distance estimation, was evaluated in real-world field trials as described in Section 2.2.2 above. The evaluation showed that the on-board obstacle detection system fulfilled these functional requirements: (1) frontal obstacle detection—detection of objects on and near the rail tracks ahead of the train which were not part of the railway infrastructure, and which could be a hazard to the train such as humans, vehicles, and large animals; (2) Detection of objects in the train’s path under different light and weather conditions. Two types of evaluation field tests were conducted: static and dynamic. In the static field tests, the vision OD sensors were mounded on a static test stand, and, in the dynamic field tests, the sensors were integrated into a sensors’ housing mounted on the front of a locomotive pulling an operational train. In both the static and dynamic field tests, the accuracy of the proposed system was evaluated through the estimated object distance between the object and the camera (static in case of static filed tests, moving in the case of dynamic field tests). The evaluation metric in both cases was the relative error with respect to the ground truth object distance.

In [65], the world’s first commercially available anti-collision system for light rail vehicles ODAS (Obstacle Detection Driver Assistance System for tramways) was presented, which was developed by a team led by Bombardier Transportation (BT). The obstacle detection is based on based on stereo-vision. The evaluation by dedicated automatic detection analysis tools and drivers’ feedback over three years and thousands of accumulated kilometres in real-world operational environment showed that the great majority of real obstacles presenting collision risks have been detected. However, evaluation also showed that the occurrence of false positives, where ODAS detects obstacles that do not present real collision risk, was still sometimes too high, as the occurrence of FP was often above a specified “comfort” limit for FP of 10% of total number of detections. The main evaluation methodology was a comparison of the performance of the ODAS system and visual performance of human drivers with respect to obstacle detection; it was concluded that the ODAS system’s performance and limitations in terms of visibility were similar to that of the driver’s eyes. In addition, the drivers’ feedback was positive as the TP detections help the driver focus their attention on the driving task.

## 4. Discussion, Identifying Research Gaps and Future Research Directions

Obstacle detection in railways refers to the detection of objects on the rail tracks ahead of a running train that are potentially hazardous. Bearing this definition in mind, it is understandable that OD systems in railways mainly consist of two consecutive steps: detection of rail tracks for the purpose of detection of image region of interest (ROI), and detection of objects in and near the ROI. Initial work on OD in railways followed previously developed and established work on obstacle detection in the automotive field. However, this approach had only limited effectiveness as the OD methods in railways had to meet the specific challenges of the railway environment such as having the train’s path fixed to the rail tracks whose appearance significantly changes in different illumination and weather conditions and long braking distance of a train which demands long-range obstacle detection.

Traditional CV-based methods for rail tracks and obstacles detection used “hand-crafted” features. However, appearance of these features changes significantly in the images captured in different environmental conditions, making the conventional CV-based methods not robust to such external influences. In more recent AI-based methods, this problem is overcome, as the “hand-crafted” features used in traditional CV-based methods are replaced by learned features which are obtained by training CNNs. Generally speaking, the AI-based methods published so far are based on re-training/modification of DL-based methods already established for object recognition in other applications. These methods, however, were not explicitly designed to cover railway specific requirements such as accurate and timely detection of distant objects on or near the rail tracks.

Furthermore, the applicability of published approaches for AI-based obstacle detection methods to railway applications is difficult to evaluate because of the absence of railway specific datasets. It is well known that public datasets have greatly contributed to progress in obstacle detection technologies in the automotive field. Individual authors in the railway field used data available from the Internet or developed custom made datasets for training and testing their own AI-based methods. However, these datasets do not cover use-cases relevant in railways and are missing standardized scenarios, making it impossible to benchmark different methods. Hence, for railway applications, datasets covering the full range of railway scenarios are needed, to allow rigorous evaluation of existing methods and to stimulate new research directions. With the current expansion of research and development of OD systems for railways, within the broader context of digitalization of rail transport, it is expected that relevant datasets recorded in real-world environments will be available soon. However, it is likely that such datasets will be biased to some objects classes likely to appear in railway scenes such as cars and pedestrians while lacking rarer objects such as fallen trees and rocks that nonetheless represent threats to safe train operation. In this regard, a promising research opportunity is the development of methods for augmenting datasets with synthetic images that represent all obstacles of all relevant classes. A method in this research direction was already presented in [69]. This method included the generation of synthetic images of railway intruding object (potential obstacles) based on an improved conditional deep convolutional generative adversarial network (C-DCGAN), in order to overcome the absence of potential obstacles in image data sets gathered from real railway operations. To investigate the authenticity and quality of the generated intruding objects, the generator, multi-scale discriminators, and a novel loss function of the improved C-DCGAN model were constructed. An intruding-object scales estimation algorithm based on a rail track gauge constant was used to generate and add synthetic intruding objects with accurate scaling into railway scene images taken from surveillance videos along high-speed rail lines. The object samples (classes human, horse, cow and sheep), which were used for generating these synthetic objects, were extracted from semantic object labels of publicly available data sets. The experimental results on the created dataset demonstrated that the synthetic object images were of a high quality, diversity, and scale accuracy, and could be used for the training and testing of the intruding object detection algorithm. However, the presented method augmented the dataset with images from static surveillance cameras, which limits its application for on-board obstacle detection, and also considers only “usual object classes” and not those specific for railway applications such as fallen trees and rocks. Considering the specific case of fallen trees, their automated detection by AI-based methods is currently problematic, or even impossible, mainly because of the underrepresentation of this scenario in the training dataset. A possible solution could be the development of a synthetic dataset with images showing fallen trees on rail tracks. However, a problem with this synthetic datasets could be that the target domain and the source domain (domain of the synthetically added object) are different which would cause that the performance of the object detection network does not achieve a sufficiently high reliability. In [70], a very promising approach is presented that proposes an objective function with several weakly-supervised constraints to learn a deep semantic segmentation network. To reduce the negative effect of the shift from source to target domain, a DualGAN (dual Conditional Generative Adversarial Network) is used, which is able to perform unsupervised style transfer between source and target domain. Following this style transfer, the created images can then be added to the training dataset for the target domain.

Another potential research direction is the improvement of obstacle distance estimation. As described in Section 2.1.3 and Section 2.2.3 above, the majority papers published so far published papers on OD in railways do not explicitly discuss estimation of distances between individual detected obstacles and on-board cameras, although this is recognized as an important function. To be useful in the railway application, future work in the field should increase the focus on obstacle distance estimation, particularly in the long range, as this is an essential component of autonomous obstacle detection that will be needed in the autonomous trains of the future. More specifically, the development of the deep-learning based methods with end-to-end frameworks to directly predict distances for given objects in the images could be explored to overcome the limitations of current vision-based distance estimation methods, with particular focus on objects distant from the on-board cameras or on curved sections of track.

## 5. Conclusions

To authors’ knowledge, this paper presents the first comprehensive literature review of systems for obstacle detection and distance estimation in railways, which use on-board vision sensors (cameras). The paper classifies all methods for vision-based on-board estimation in two categories: traditional CV-based methods and AI-based methods. For each category, three essential aspects are analyzed: rail track extraction, detection of obstacles on rail tracks and estimation of distances between on-board cameras (train) and detected obstacles. In addition, reported evaluation tests are analyzed in the paper, and they are classified as evaluation on images/videos available in Internet and evaluation on real-world images/videos. The second group can be divided into two sub-groups: evaluation on custom images/videos modified offline by adding digital objects on the videos and evaluation on images/videos recorded in real-world operational conditions. The paper finishes with discussion, critiques, and perspective on vision-based obstacle detection and distance estimation in railways. Finally, some promising research topics for OD in railway have been proposed that could advance system overall performance and in particular improve distance estimation.

## Figures and Tables

**Table 1:** Summary of the papers describing traditional CV methods.

Paper	Type of Vision System	Rail Track Detection	Obstacle Detection	Distance Estimation	Evaluation Tests—Comments
Fel et al., 2018 [65]	Stereo-vision system consisting of 3 RGB cameras	✓	✓	Short-range obstacle detection using stereo cameras with a small stereo baseline (0.25 m) and long range depth estimation with a larger baseline (0.8 m)	The evaluation from dedicated automatic detection analysis tools and the drivers’ feedbacks over three years and thousands of accumulated kilometres
Fioretti et al., 2018 [40]	Monocular RGB on-board cameras	✓	✓	3D reconstruction of rails and of objects such as kilometres’ signs; distance (z-coordinate) range up to 200 m	Evaluation on image data of a single track railway recorded for 8 min and 20 s for 9 km at train speed of about 72 km/h.
Fonseca Rodriguez et al., 2012 [41]	—	✓	✓	—	Evaluation on several modified videos from the Internet; digital objects artificially added to video frames
Gavrilova et al., 2018 [45]	Standard RGB on-board camera	✓	✓	Using the image coordinates of the last point on detected obstacle-free rail tracks as wagon point coordinates, to calculate actual distance to wagon; up to 100 m	Evaluation on real-world images; 100 images with the object (wagon) on the rail tracks and 300 images without the object
Gebauer et al., 2012 [7]	Stereo-cameras + thermal camera	✓	✓	Using stereo-vision and laser scanner; up to 80 m	Evaluation on real-world images recorded on 15 km long railway in Austria on which a prototype train operates
Gschwandtner et al., 2010 [37]	Grayscale camera	✓	—	—	Evaluation on a dataset acquired in winter on the track length of approx. 15 km and the train driving at 40 km/h.
Kaleli et al., 2009 [34]	Standard RGB camera	✓	—	—	Evaluation on several video sequences taken from the on-board camera while the train was moving at high speeds
Kudinov et al., 2020 [31]	Grayscale camera	✓	✓	Short-range distances to wagons up to 50 m (absolute error no more than 1.2 m)	Evaluation on real-world images of the shunting track of the Ryazan-1 railway station recorded in cloudy weather
Maire et al., 2010 [33]	Grayscale camera	✓	—	Detection of obstacle-free rail tracks zone of about 100 m but no individual object distance estimation	Evaluation on real-world images of rather poor quality
Möckel et al., 2003 [16]	Multi-focal camera system, one short-range and two long-range cameras	✓	✓	Using LiDAR with an up to 400 m look-ahead range but no individual object distance estimation	Evaluation on images from real-world field tests on a public rail track near Munich used as test track; simulated obstacles
Mukojima et al., 2016 [42]	Standard RGB camera	—	✓	—	Evaluation on train frontal view images captured on a test line in the premises of the Railway Technical Research Institute, Japan.
Nakasone et al., 2017 [22]	Standard RGB camera	—	✓	The farthest obstacle at 235 m as the test track was straight at a length of about 250 m leading to the obstacle	Images from four train runs without obstacles used as reference. Evaluation on 5000 frames with obstacles captured in 17 train runs
Nassu et al., 2011 [27]	Standard RGB camera	✓	—	Rail extraction in the long distance was performed in addition to rail extraction in the short distance; however, the long distance range was not defined	Three sets of videos captured in real operating conditions in several Japanese railways. The 1st set has 10 videos, with 3549 frames, the 2nd has 27 videos, with 14,474 frames and the 3rd has 12 videos, with 5879 frames with rails are mostly visible in the long distance
Nassu et al., 2012 [26]	RGB camera with zoom lens mounted on a pan–tilt unit	✓	—	Different rail exraction methods for short distances and long distances; no specification on distance range	Evaluation on 12 video sequences captured under real operating conditions in several Japanese railways, with 459,733 frames (4:15 h of recording).
Qi et al., 2013 [21]	RGB camera	✓	—	—	Evaluation on six real-world video datasets recorded in different illumination. The test data were collected in Hefei City, Anhui Province of China. The length of the rails approx. 10 km. The train speed of about 60 km/h.
Ross 2010 [19]	Grayscale camera	✓	—	—	Evaluation on on-board camera images taken near Karlsruhe, Germany. Dataset included images of single track situations and of turnout situations
Saika et al., 2016 [66]	Standard RGB camera	—	✓	—	Evaluation on a train driver’s view video, of scene of train approaching a platform, available on the Internet. The test video sequences of 12 frames
Selver et al., 2016 [38]	—	✓	—	—	Evaluation data set was a collection of publicly available in Internet cabin view videos (different weather conditions); 389 frames
Ukai, 2004 [29]	Ultra telephoto lens camera	✓	✓	Used camera allows for monitoring the rail tracks ahead up 600 m but no individual object distance estimation	The image sequence of 557 frames (18.56 s) recorded from a train was collected for evaluation
Uribe et al., 2012 [17]	—	✓	✓	—	Evaluation on images from Internet with artificially added obstacles
Vazquez et al., 2004 [43]	RGB camera	—	✓	—	Evaluation on real images captured in railway environment and in varied illumination and weather conditions (some images in very adverse conditions as images with fog)
Wang et al., 2017 [32]	Standard RGB on-board camera	✓	✓	—	Evaluation dataset is an open source video captured by on-board camera from Malmo to Angelholm, Sweden; videos were modified adding digital obstacles of different nature and shape
Wang et al., 2015 [35]	Grayscale camera	✓	—	—	Test images were taken at Fengtai west railway station freight yard, Beijing
Wang et al., 2016 [36]	Grayscale camera	✓	—	—	Test images were taken at Fengtai west railway station freight yard, Beijing
Weichselbaum et al., 2013 [12]	Stereo-vision	✓	✓	Obstacle distance from 10 m up to 80 m ahead	Evaluation on two representative real-world test sequences with various numbers of frames, situations and obstacles
Wohlfeil, 2011 [23]	Grayscale camera	✓	—	—	Evaluation test images from six different test rides in three different places at German railway tests sites; various challenging lightning conditions
Yamashita et al., 1996 [8]	Mono thermal camera with a telephoto lens	✓	✓	Using laser range finder, up to 1 km	Real-world tests; the system on-board test train of the East Japan Railway

**Table 2:** Summary of the papers describing AI-based methods.

Paper	Type of Vision System	Rail Track Detection	Obstacle Detection	Distance Estimation	Evaluation Tests—Comments
Haseeb et al., 2018 [60,67]; Haseeb et al., 2019 [68]; Ristić-Durrant et al., 2020 [61]	RGB cameras + thermal camera + night vision camera	—	✓	Feedforward neural network (NN)-based distance estimation. NN estimates object distance based on the features of the object Bounding Box (BB) extracted by the DL-based object detector. Mid-range (80–200 m) and long-range (up to 1000 m) distance estimation	Real-world tests; on-board multi-camera system mounted on the operational locomotive owned by Serbia Cargo and running on Serbian part of pan-European Corridor X in length of 120 km in different illumination and weather conditions
Wang et al., 2019 [18]	Standard RGB on-board camera	✓	—	—	Evaluation on 300 images recorded in operational environment of low-speed autonomous trains in China
Wang et al., 2018 [48]	Standard RGB on-board camera	✓	—	—	Evaluation on 1123 images recorded on the Beijing metro Yanfang line and Shanghai metro line 6 including tunnels and open lines
Wedberg, 2017 [53]	Thermal on-board camera	—	✓	Detecting rails at long-range; no details of distance range	The evaluation dataset consisted of on-board thermal infrared video sequences. The data were collected in northern Sweden in April 2015
Xu et al., 2019 [59]	Standard RGB HD camera	—	✓	—	Evaluation on 1277 images recorded on the Hongkong MTR Tsuen Wan Line, Beijing Metro Yanfang line and Shanghai metro line 6 in different conditions, in daytime and night, sunny and rainy days
Ye et al., 2018 [13]	Standard RGB camera	✓	✓	Using millimeter-wave radar; no details on object distance measurement	Evaluation of custom dataset recorded in real-world railway shunting scenarios
Ye et al., 2020 [14]	Standard RGB camera	✓	✓	Using millimeter-wave radar; no details on object distance measurement	Evaluation of custom dataset recorded in real-world railway shunting scenarios
Ye et al., 2020 [25]	Standard RGB camera and a near infrared laser, supplementing the illumination of the camera	✓	✓	—	Evaluation of custom dataset recorded in real-world railway shunting scenarios
Yu et al., 2018 [54]	—	—	✓	—	Evaluation on 5000 images of outdoor railway scenes, which were collected from the Internet

**Table 3:** Summary of the papers describing hybrid systems (traditional CV/AI-based methods).

Paper	Type of Vision System	Rail Track Detection	Obstacle Detection	Distance Estimation	Evaluation Tests—Comments
Chernov et al., 2020 [64]	Stereo RGB cameras	✓	✓	Stereo-vision system enables determining the safe distance from the yard locomotive to the car coupling device; no details on distance estimation	Evaluation on real-world test images recorded by stereo cameras mounted on yard locomotives
Kapoor et al., 2020 [56]	Thermal camera	✓	✓	Used cameras range up to 1500 m; Detection of objects within the rail tracks portions visible in the image but no individual object distance estimation	Evaluation on 749 images recorded with on-board thermal camera; no details of evaluation tests
Selver et al., 2017 [39]	Standard RGB camera	✓	—	—	Evaluation on 2185 manually delineated frames. These are obtained from 29 left and 24 right turns belonging acquired during common public journeys.
